# Prevalence and multilocus genotyping of *Giardia duodenalis* in Holstein cattle in Yunnan, China

**DOI:** 10.3389/fvets.2022.949462

**Published:** 2022-10-21

**Authors:** Zhao-Jun Heng, Jian-Fa Yang, Xin-Yan Xie, Cui-Rong Xu, Jun-Rong Chen, Jun Ma, Jun-Jun He, Hua-Ming Mao

**Affiliations:** ^1^Key Laboratory of Veterinary Public Health of Yunnan Province, College of Veterinary Medicine, Yunnan Agricultural University, Kunming, China; ^2^College of Animal Science and Technology, Yunnan Agricultural University, Kunming, China

**Keywords:** *Giardia duodenalis*, prevalence, multilocus genotypes (MLGs), Holstein cattle, genotyping

## Abstract

*Giardia duodenalis* is an important zoonotic protozoon, which can infect a variety of animals, causing diarrhea and even death of animals or humans. Dairy cattle have been implicated as important sources of human *G. duodenalis*. However, the information about the prevalence and genetic diversity of *G. duodenalis* in dairy cattle in China's Yunnan Province remains limited. This study investigated the occurrence and multilocus genotyping of *G. duodenalis* of Holstein cattle in Yunnan Province, China. A total of 524 fresh fecal samples of Holstein cattle were randomly collected from 8 farms in Yunnan. In this study, 27.5% (144/524) of tested samples were positive for *G. duodenalis* infection. The highest infection ratio was found in preweaned calves (33.7%), and the infection rates of postweaned calves, growing cattle, and adult cattle were 24.5%, 23.0%, and 17.3%, respectively. The sequence analysis of SSU rRNA gene showed that the predominant assemblage of *G. duodenalis* in this study was assemblage E (97.9%, 141/144), whereas assemblage A was identified only in three samples (2.1%, 3/144). All *G. duodenalis*-positive samples were further assayed with nested polymerase chain reaction (PCR) targeting β-giardin (bg), triosephosphate isomerase (tpi), and glutamate dehydrogenase (gdh) genes, and 87, 41, and 81 sequences were obtained, respectively. Mixed infection of assemblages A and E of *G. duodenalis* was detected in three samples. Multilocus genotyping yielded 23 multilocus genotypes (MLGs). This is the first study that reveals the prevalence data of *G. duodenalis* in Holstein cattle in Yunnan Province, and the results of this study provided baseline data for the prevention and control of *G. duodenalis* infection in Holstein cattle in Yunnan Province, China.

## Introduction

*Giardia duodenalis* is one of the most common parasitic protists that can infect humans, livestock, companionn animals, and wildlife (1). *G. duodenalis* has a simple life cycle that consists of two stages of development (trophozoite and cyst) (2–4). Trophozoite is the replicative stage that can cause clinical symptoms of giardiasis, while cyst is the main stage of infection (5, 6), and the cysts can excyst in the small intestine when exposed to bile salts and gastric acid. One *G. duodenalis* cyst releases two trophozoites that parasitize the intestinal epithelia of duodenum and jejunum. Cysts are environmentally resistant and they can survive at 0–8°C for 2 months. Successful infection of *G. duodenalis* can be established by ingestion of 10–25 cysts (2, 7). *G. duodenalis* could lead to acute or chronic diarrhea, abdominal cramps, nausea, vomiting, weight loss, and malabsorption in the infected hosts (8, 9), and the severity of clinical symptoms of Giardiasis is related to the virulence of the genotype of *G. duodenalis* (10).

*G. duodenalis* consists of eight assemblages (A–H), and some of those aggregates display host specificity (6, 11). Assemblages A and B of *G. duodenalis* infect various mammals (e.g., bovines) (7), assemblages C and D infect dogs and other canines, assemblage E infects hoofed animals, assemblage F infects cats, assemblage G infects rodents, and assemblage H infects marine vertebrates (1, 12). Recently, assemblages C, D, E, and F have also been found in humans (13–16). Dairy cows are dominantly infected with *G. duodenalis* of assemblages A, B, and E (11, 17). Calves are more frequently infected with zoonotic assemblages A and B than adult cattle (18). *G. duodenalis* is one of the most important parasitic pathogens that causes calf diarrhea (19). The infection rate of *G. duodenalis* in cattle ranges from 2 to 89% (20–27). There was a relatively high prevalence of *G. duodenalis* infection in cattle in China (28–36). Humans and other animals can be infected by ingesting food or water contaminated with *Giardia* cysts (8, 37). It is clear that cattle is an important zoonotic reservoir of *G. duodenalis* and plays important roles in the cross transmission between humans and cattle (38, 39).

A previous study has shown that the occurrence of mixed infection with different assemblages is common in animals (1). Since the use of multiple markers can obtain more reliable results for genotyping (1, 40, 41), multilocus sequence typing (MLST) or multilocus genotypes (MLGs) has been widely applied to study the population genetic structure of parasites, detecting and discriminating the mixed infections of different assemblages (or subassemblages) (42, 43). SSU rRNA, β-giardin (bg), triosephosphate isomerase (tpi), and glutamate dehydrogenase (gdh) genes are four commonly used genetic markers in the genotyping of *G. duodenalis*. Mixed infection of *G. duodenalis* will result in inconsistent genotyping results of different loci for that bg, tpi, and gdh genes show high genetic polymorphism (41).

Up to now, the infection data of *G. duodenalis* in dairy calves mainly focus on the difference between preweaned and postweaned stages in China. The prevalence data of *G. duodenalis* in dairy cattle remain to be limited in Yunnan, especially the molecular data. In this study, we investigated the infection of *G. duodenalis* in Holstein cattle in some areas of Yunnan Province by using nested polymerase chain reaction (PCR) targeting the small subunit ribosomal RNA (SSU rRNA) gene of *G. duodenalis*. All positive samples were further subjected to the gene analysis of bg, tpi, and gdh genes for the genotyping of *G. duodenalis*.

## Materials and methods

### Ethical statements

This study was approved by the Life Science Ethics Committee of Yunnan Agricultural University with the ethical code 202109003. Fecal samples were collected from the Holstein cattle with the permission of the farm owners or managers.

### Sample and data collection

From July to November 2021, a total of 524 fecal samples of Holstein cattle were randomly collected from 8 farms, including one free-ranging farm and seven intensive feeding farms. The age of Holstein cattle ranged from newborn to 2 years old. The collection sites included Dali, Kunming, Qujing, and Chuxiong. Feces were collected from 422 females and 102 males, and only 18 of them had obvious clinical symptoms of diarrhea. Fresh fecal samples (10–20 g per cattle) were collected directly from the rectum using disposable gloves and then transferred separately into disposable plastic bags, marked with the date, age, and geographical information. Fecal samples were stored at 4°C until used for DNA extraction.

The age of Holstein cattle was classified according to the Technical Specification for Standardized Scale Breeding and Production of Dairy Cows (Trial) issued by the Ministry of Agriculture of the People's Republic of China. The cattle ≤ 60 days old are preweaned calves, 61–180 days old are postweaned calves, 181–450 days old are growing cattle, and ≥ 450 days old are adult cows.

### DNA extraction and PCR amplification

Before DNA extraction, stored feces were washed with distilled water and centrifuged at 3,000 × *g* for 3 min; 250 mg of each washed sample was used for DNA extraction individually. The DNA of the collected sample was extracted by using E.Z.*N*.A.R^®^ Stool DNA Kit (Omega Bio-Tek Inc., Norcross, GA, USA) according to the manufacturer's instructions. The extracted DNA was stored at −20°C until use.

All DNA samples were tested with nested PCR that targets the SSU rRNA of *G. duodenalis* to determine the infection of *G. duodenalis* (44). The bg (45), tpi (46), and gdh (41) genes were used to determine the subtypes of *G. duodenalis*. The primers, annealing temperatures, and the expected product sizes of nested PCR are listed in Table 1. The nested PCR reaction of SSU rRNA, bg, gdh, and tpi loci was conducted in a 25-μl reaction system containing 10 × PCR buffer, 200 μM of each dNTP, 0.4 μM of each primer, 1 unit of TaKaRa r-*Taq* DNA polymerase (TaKaRa Shuzo Co., Ltd.), and 2 μl of DNA sample. Dimethyl sulfoxide (DMSO) was added to enhance the amplification efficiency of nested PCR. The products of the second nested PCR were subjected to electrophoresis on 1% agarose gel and photographed by using a gel imaging system.

**Table 1:** Primers and expected amplicon sizes for PCR detection and amplification.

**Gene**	**Primer (sequence 5^′^-3^′^)**	**Fragment length** ** (bp)**	**Annealing temperature** ** (°C)**	**References**
SSU r RNA	GIA20: AAGTGTGGTGCAGACGGACTC	292	55	(44)
	GIA21: CTGCTGCCGTCCTTGGATGT			
	RH11: CATCCGGTCGATCCTGCC		59	
	RH4: AGTCGAACCCTGATTCTCCGCCCAGG			
bg	G7F: AAGCCCGACGACCTCACCCGCAGTGC	511	50	(45)
	G7R: GAGGCCGCCCTGGATCTTCGAGACGAC			
	GF: GAACGAACGAGATCGAGGTCCG		60	
	GR: CTCGACGAGCTTCGTGTT			
tpi	ALF1: AAATIATGCCTGCTCGTCG	530	58	(46)
	ALR1: CAAACCTTITCCGCAAACC			
	ALF2: CCCTTCATCGGIGGTAACTT		62	
	ALR2: GTGGCCACCACICCCGTGCC			
gdh	Gdh1: TTCCGTRTYCAGTACAACTC	530	50	(41)
	Gdh2: ACCTCGTTCTGRGTGGCGCA			
	Gdh3: ATGACYGAGCTYCAGAGGCACGT		65	
	Gdh4: GTGGCGCARGGCATGATGCA			

### DNA sequencing

The positive secondary nested PCR products were sent to Shenggong Bioengineering (Shanghai) Co., Ltd. for bidirectional sequencing. All sequences obtained in this study were searched against GenBank by using BLAST, and the Clustal X software was used for sequence alignment analysis. All representative nucleotide sequences generated at bg, tpi, and gdh loci in this study were deposited in the GenBank database under accession numbers ON773555–ON773581.

### Statistical analysis

The chi-square (χ^2^) test was used to analyze the differences in *G. duodenalis* infection in Holstein cattle among different regions, age, sex, and farming methods. The confidence interval was set as 95%, and *P* < 0.05 was considered statistically significant. All statistical analyses were performed by using the SPSS20.0 statistical software.

## Results

### Prevalence and risk factors of *Giardia duodenalis*

In this study, 144 samples were *G. duodenalis* positive, and the global positive ratio of *G. duodenalis* was 27.5% (144/524) (Table 2). *G. duodenalis* infection was found in all 8 farms, while the prevalence of *G. duodenalis* varied from farm to farm, and the infection rate ranged from 5.6 to 43.7% (Table 2). The highest infection rate was found in Dali (44.0%, 40/91), followed by Shilin (40.4%, 23/57) and Wuding (40%, 4/14). The lowest infection rate was found in Qijiashan Ranch (5.6%, 5/89) in Qujing. This study showed that the prevalence of *G. duodenalis* among different regions was significantly different (χ^2^ = 57.74, df = 8, *P* < 0.01). Infection of *G. duodenalis* was found in Holstein cattle in all age groups, among which the highest infection rate (33.7%) was found in preweaned calves. Adult cattle showed the lowest infection rate (17.3%). The χ^2^ test showed that the prevalence of *G. duodenalis* among the four age groups was significantly different (χ^2^ = 11.56, df = 3, *P* < 0.01) (Table 3). No significant difference was found between intensive feeding and free-ranging farms (χ^2^ = 2.95, df = 1, *P* > 0.05) (Table 3). By comparing the infection in different sexes, we found that the infection rate in females and males was 25.6% and 35.3%, respectively. The difference was significant between female and male (χ^2^ = 3.88, df = 1, *P* < 0.05) (Table 3). In addition, in this study, no significant difference was found between diarrhea sample (22.2%) and normal feces (27.7%) (χ^2^ = 0.26, df = 1, *P* > 0.05) (Table 3).

**Table 2:** Occurrence of *Giardia duodenalis* in Holstein cattle in Yunnan Province.

**Sampling site**		**Simple size**	**Number of positive specimens**				**No. positive (%)**	**Odds ratio (95% CI)**	***P***
			**SSU**	**bg**	**tpi**	**gdh**			
Dali	Ouya	40	10	6	2	8	25.0	5.6 (1.8–17.7)	<0.01
	Juxin	29	4	2	0	1	13.8	2.7 (0.7–10.8)	
	Heqing	114	27	20	13	20	23.7	5.2 (1.9–14.2)	
	Dali	91	40	31	14	27	44.0	13.2 (4.9–35.6)	
Kunmin	Shilin	57	23	13	6	14	40.4	11.4 (4.0–32.3)	
Qujing	Qijiashan	89	5	2	0	0	5.6	Ref. group	
	Luliang	69	21	7	2	5	30.4	7.4 (2.6–20.7)	
Chuxiong	Wuding	35	14	6	4	6	40	11.2 (3.6–34.6)	
Total		524	144	87	41	81	27.5		

**Table 3:** Risk factors of *Giardia duodenalis* in Holstein cattle.

**Risk factors**		**Sample size**	**No. positive (%)**	**OR (95% CI)**	***P***
Age (d)	<60	258	87 (33.7)	2.4 (1.4–4.3)	0.009
	61~180	143	35 (24.5)	1.6 (0.8–2.9)	
	181~450	13	3 (23)	1.4 (0.4–5.7)	
	>450	110	19 (17.3)	Ref. group	
Sex	Female	422	108 (25.6)	Ref. group	0.049
	Male	102	36 (35.3)	1.6 (1.0–2.5)	
Farming model	Intensive farming	489	130 (26.6)	Ref. group	0.086
	Free–ranging	35	14 (40)	1.8 (0.9–3.7)	
Clinical symptom	Asymptomatic	506	140 (27.7)	1.3 (0.4–4.1)	0.6
	Diarrhea	18	4 (22.2)	Ref. group	

### Molecular identification and polymorphisms of *Giardia duodenalis* isolates

Sequence analyses of the amplified 144 SSU rRNA genes showed that three of them were classified into *G. duodenalis* assemblage A (2.1%, 3/144) and the rest of them were a member of assemblage E (97.9%, 141/144). Of the 144 positive fecal samples, 60.4% (*n* = 87) were bg positive, 28.5% (*n* = 41) were tpi positive, and 56.3% (*n* = 81) were gdh positive. Notably, 20.1% (*n* = 29) of samples were positive for all four genes in this study.

The bg subtype analysis showed that 9 subtypes of assemblage E and 1 subtype of assemblage A were identified, among which 5 subtypes of assemblage E had previously been identified; for that, there was a 100% similarity to the sequences available in GenBank with accession numbers of E9 (KY769091, *n* = 8), E3 (MK252653, *n* = 7), E5 (KY769092, *n* = 6), E1 (MK252651, *n* = 2), and E2 (MK252652, *n* = 1); the remaining 4 subtypes represent novel subtypes (Table 4). Assemblage A shares the same sequence with MK610391 in GenBank. Of the tpi subtype, 16 subtypes of assemblage E and 2 subtypes of assemblage A were observed, including 3 subtypes of assemblage E and 1 subtype of assemblage A with sequences identical to those in GenBank, namely, E34 (MK252659, *n* = 9), E17 (MK252661, *n* = 3), E2 (EF654683, *n* = 1), and A1 (MK639171, *n* = 2). The remaining 13 subtypes of assemblage E and 1 subtype of assemblage A represented novel sequences. At the gdh locus, 13 subtypes of assemblage E and 1 subtype of assemblage A were identified, including 5 known subtypes of assemblage E with sequences identical to those in GenBank, namely, E3 (KY769099, *n* = 9), E1 (KY769096, *n* = 8), E14 (KY769097, *n* = 1), E15 (KY432839, *n* = 1), and E (MH794177, *n* = 1). The remaining 8 subtypes of assemblage E and 1 subtype of assemblage A represented novel subtypes in this study (Table 4). Of these subtypes, E9 (*n* = 8, bg subtype), E34 (*n* = 6, tpi subtype), and E3 (*n* = 8, gdh subtype) were the predominant subtypes.

**Table 4:** Multilocus sequence genotypes of *Giardia duodenalis* assemblage E in Holstein cattle in Yunnan Province.

**Isolate**	**Genotype (GenBank accession no.)**			**MLG type (*n*)^a^**
	**bg**	**tpi**	**gdh**	
OY14	E9 (KY769091)	E2 (EF654683)	E3 (KY769099)	MLG E1 (1)
OY17	E5 (KY769092)	E36^b^	E3	MLG E2 (1)
HQ24	A1 (MK610391)	A10^b^	E36^b^	Mixed 1
HQ30	E3 (MK252653)	E37^b^	E37^b^	MLG E3 (1)
HQ31	E5	E38^b^	E1 (KY769096)	MLG E4 (1)
HQ45	E5	E39^b^	E1	MLG E5 (1)
HQ71	E9	E40^b^	E3	MLG E6 (1)
HQ84	E9	E41^b^	E14 (KY769097)	MLG E7 (1)
HQ87	E5	E34 (MK252659)	E1	MLG E8 (1)
HQ90	E9	E42^b^	E3	MLG E9 (1)
HQ93	E9	E43^b^	E38^b^	MLG E10 (1)
HQ102	E9	E44^b^	E3	MLG E11 (1)
DL9, 14, 79	E3	E34	E1	MLG E12 (3)
DL12	E9	E17 (MK252661)	E1	MLG E13 (1)
DL24	E36^b^	E45^b^	E15 (KY432839)	MLG E14 (1)
DL26	E37^b^	E46^b^	E39^b^	MLG E15 (1)
DL48, 81	E3	E34	E3	MLG E16 (2)
DL67	E3	E34	E40^b^	MLG E17 (1)
DL71	E9	E17	E41^b^	MLG E18 (1)
DL84	E38^b^	E34	E1	MLG E19 (1)
SL16	E1 (KY769095)	E34	E (MH794177)	MLG E20 (1)
SL37	E39^b^	A1	E42^b^	Mixed 2
SL38	E2 (MK252652)	E47^b^	E3	MLG E21 (1)
SL46	E1	A1	AI10^b^	Mixed 3
WD22	E5	E48^b^	E3	MLG E22 (1)
WD28	E5	E17	E43^b^	MLG E23 (1)

a MLG in this study.

b Novel subtype in this study.

In this study, 29 samples were simultaneously amplified at all three intra-assemblage variation genetic loci. Notably, 26 samples' *G. duodenalis* belonged to assemblage E, including 23 novel assemblage E MLGs (named MLG-E1 to MLG-E23). Three samples showed mixed infection with assemblages E and A (Table 4). MLG-E1 and MLG-E2 were the predominant MLGs in this study. MLGs were detected only in preweaning calves and postweaning calves.

## Discussion

This study indicated that 27.5% of the tested Yunnan Holstein cattle were infected with *G. duodenalis*, which is similar to the result of dairy cows reported in Hubei (22.6%, 77/339) (33). The infection rate of *G. duodenalis* (27.5%) observed in this study was higher than that of most provinces in China, such as Jiangsu (20.6%, 281/1,366) (34), Henan (7.2%, 128/1,777) (35), Xinjiang (13.4%, 69/514) (36), and Hebei and Tianjin (4.7%, 49/1,040) (47). It was also higher than that of yak in Qinghai (2.04%, 21/1,027) (48), Tibetan cattle in Tibet (3.8%, 17/442 ) (49), and Yunling cattle in Yunnan (10.49%, 41/391) (30) but lower than that of calf in Sichuan (41.2%, 26/306) (50), Guangdong (74.2%, 288/388) (31), and Shanghai (60.1%, 492/818) (32). Compared with *G. duodenalis* infection in other countries, the overall infection rate in this study was higher than that of Thailand (5.0%, 45/900) (51), South Korea (5.6%−12.7%) (52, 53), Iran (4.2%, 8/192) (54), and Egypt (13.3%, 33/248) (55). Infection rates are affected by a series of factors, such as geographical and ecological conditions, animal age, health status, sampling season, and diagnostic and research methods.

Previous studies have revealed that the prevalence of *G. duodenalis* is associated with animal age (35, 56, 57), and the infection is inversely associated with animal age (8, 58, 59). As shown in Table 3, the prevalence of preweaned calves in this study was significantly higher than that of postweaned calves, and the infection rate of *G. duodenalis* gradually decreased with the increase of age. The finding of this study is consistent with previous reports (34, 60–62). It could be the result that calves are more susceptible to *G. duodenalis* than adult cows. In this study, no significant difference was observed between intensive feeding and free-ranging farms (χ^2^ = 2.95, df = 1, *P* > 0.05), which is consistent with the findings of a previous study in Sichuan (50). By comparing the infection ratio between different sexes, the results of this study showed that the infection rate in females was 25.59%, while the infection rate in males was 35.29%, which is statistically significant (χ^2^ = 3.88, df = 1, *P* < 0.05) and contrary to the prevalence data of *G. duodenalis* in Hubei (33). *G. duodenalis* infection showed no significant correlation between the stool sample types in this study. This result agreed with the findings of the previous study in Korea (63), although some studies showed that there was a statistical association between *G. duodenalis* and the type of fecal sample (53).

At present, a total of 8 assemblages (A–H) have been found in *G. duodenalis*. In this study, two assemblages (assemblages A and E) were detected in dairy cows, and assemblage E was the dominant assemblage in this study. In other studies, assemblage E was also the predominant genotype in dairy cows (1, 32, 35, 36). It has been generally believed that assemblage E is animal-specific and mostly infects ungulates. However, the occurrence of assemblage E in human in Australia (9), Brazil (64), and Egypt (16, 65) has been reported. In this study, zoonotic assemblage A was observed in Dali and Kunming areas, and assemblage A found in this study is close to assemblage A found in the human body. These results indicate that the infected Holstein cattle of Dali and Kunming could be a potential source of zoonotic *G. duodenalis*.

In this study, SSU gene loci-positive samples were further analyzed by multilocus genes to reveal genetic variation in *G. duodenalis*. A total of 9 assemblage E subtypes and 1 assemblage A subtype were identified by using bg locus, 16 assemblage E subtypes and 2 assemblage A subtypes were identified by using tpi locus, and 13 assemblage E subtypes and 1 assemblage A subtype were identified by using gdh locus. The combination of sequence polymorphisms at these three loci led to the identification of 23 E MLGs, and three samples had different assemblages at three loci (Table 4). In this study, *G. duodenalis* A+E mixed infection was detected in preweaned and postweaned calves, which is consistent with other studies in Xinjiang (36), Henan, (35) and Shaanxi (62) provinces of China and Europe (57). All three genes were successfully amplified and sequenced from 29 isolates. A total of 23 MLGs of assemblage E and 3 MLGs of assemblage E+A were identified by three loci, among which MLG-E12 and MLG-E16 were the dominating MLGs in this study.

In this study, there was less overlap for MLGs among samples. It might be the result that *G. duodenalis* of Holstein cattle in Yunnan is rich in genetic diversity (66). In Guangdong and Sichuan Provinces of China, there was also a very high genetic diversity of assemblage E, and the three genetic loci (bg, tpi, and gdh) show high sequence polymorphism (31, 50). Assemblage E intra-assemblage genetic recombination may be the cause of high subtype diversity (41, 67). Previous studies have also shown that successful amplification rates of the gdh, bg, and tpi loci varied from 8% to 58% (68, 69). The samples that were positive for SSU showed the negative result for the other 3 genetic loci (bg, tpi, and gdh), possibly due to the limited sensitivity of PCR in testing the single-copy gene. This is probably the main limitation of this study. Despite this drawback, MLGs provide a necessary tool to identify different genetic variants within *G. duodenalis* (4). Further molecular epidemiological studies are needed to be performed to reveal the molecular characteristics of *G. duodenalis* in Holstein cattle in Yunnan Province, southwestern China.

## Conclusion

This is the first MLG characterization study of *G. duodenalis* in Holstein cattle in Yunnan Province, southwestern China. In addition, the factors associated with *G. duodenalis* infection were also analyzed. In this study, two assemblages (A and E) of *G. duodenalis* were found in Holstein cattle, and assemblage E was identified as the dominating genotype. The presence of zoonotic assemblage A in Heqing and Shilin cattle suggests their zoonotic potential. Multilocus genotyping at bg, tpi, and gdh loci revealed 23 novel assemblage E MLGs and 3 E+A mixed infection in Holstein cattle. These findings indicate that *G. duodenalis* of Holstein cattle in Yunnan is rich in genetic diversity, and the sequence of each gene locus is quite different. For the limited sensitivity of PCR, intensive study is required to reveal the molecular characteristics of *G. duodenalis* in Holstein cattle in Yunnan, and it is important to strengthen the surveillance of this parasitic disease to ensure the health of livestock and human beings.

## Data availability statement

The datasets presented in this study can be found in online repositories. The names of the repository/repositories and accession number(s) can be found in the article/supplementary materials.

## Ethics statement

The animal study was reviewed and approved by the Life Science Ethics Committee of Yunnan Agricultural University.

## Author contributions

H-MM, J-FY, and J-JH designed the study. J-FY performed fecal sample collection. Z-JH, X-YX, C-RX, and J-RC performed the molecular genetic studies. JM analyzed sequences. J-JH, J-FY, and H-MM provided laboratory supplies and revised the manuscript. Z-JH interpreted the results and wrote the manuscript. All authors have read and approved the final manuscript.

## Funding

This study was funded by the Scientific and Technological Mission of Dairy Cow Industry in Heqing County, Yunnan Province (Grant No. 202204BI090005), the Veterinary Public Health Innovation Team of Yunnan Province (Grant No. 202105AE160014), and the Yunnan Expert Workstation (Grant No. 202005AF150041).

## Conflict of interest

The authors declare that the research was conducted in the absence of any commercial or financial relationships that could be construed as a potential conflict of interest.

## Publisher's note

All claims expressed in this article are solely those of the authors and do not necessarily represent those of their affiliated organizations, or those of the publisher, the editors and the reviewers. Any product that may be evaluated in this article, or claim that may be made by its manufacturer, is not guaranteed or endorsed by the publisher.
